# The *Penicillium echinulatum* Secretome on Sugar Cane Bagasse

**DOI:** 10.1371/journal.pone.0050571

**Published:** 2012-12-05

**Authors:** Daniela A. Ribeiro, Júnio Cota, Thabata M. Alvarez, Fernanda Brüchli, Juliano Bragato, Beatriz M. P. Pereira, Bianca A. Pauletti, George Jackson, Maria T. B. Pimenta, Mario T. Murakami, Marli Camassola, Roberto Ruller, Aldo J. P. Dillon, Jose G. C. Pradella, Adriana F. Paes Leme, Fabio M. Squina

**Affiliations:** 1 Laboratório Nacional de Ciência e Tecnologia do Bioetanol (CTBE), Centro Nacional de Pesquisa em Energia e Materiais, Campinas, (CNPEM), Campinas, São Paulo, Brazil; 2 Laboratório de Espectrometria de Massas, Laboratório Nacional de Biociências (LNBio), Centro Nacional de Pesquisa em Energia e Materiais, Campinas, (CNPEM), Campinas, São Paulo, Brazil; 3 Instituto de Biotecnologia, Universidade de Caxias do Sul (UCS), Caxias do Sul, Rio Grande do Sol, Brazil; Universidade de Sao Paulo, Brazil

## Abstract

Plant feedstocks are at the leading front of the biofuel industry based on the potential to promote economical, social and environmental development worldwide through sustainable scenarios related to energy production. *Penicillium echinulatum* is a promising strain for the bioethanol industry based on its capacity to produce large amounts of cellulases at low cost. The secretome profile of *P. echinulatum* after grown on integral sugarcane bagasse, microcrystalline cellulose and three types of pretreated sugarcane bagasse was evaluated using shotgun proteomics. The comprehensive chemical characterization of the biomass used as the source of fungal nutrition, as well as biochemical activity assays using a collection of natural polysaccharides, were also performed. Our study revealed that the enzymatic repertoire of *P. echinulatum* is geared mainly toward producing enzymes from the cellulose complex (endogluganases, cellobiohydrolases and β-glucosidases). Glycoside hydrolase (GH) family members, important to biomass-to-biofuels conversion strategies, were identified, including endoglucanases GH5, 7, 6, 12, 17 and 61, β-glycosidase GH3, xylanases GH10 and GH11, as well as debranching hemicellulases from GH43, GH62 and CE2 and pectinanes from GH28. Collectively, the approach conducted in this study gave new insights on the better comprehension of the composition and degradation capability of an industrial cellulolytic strain, from which a number of applied technologies, such as biofuel production, can be generated.

## Introduction

Plant structural polysaccharides are the most abundant and renewable biomass in the biosphere. Plant feedstocks are at the leading front of the biofuel industry based on the potential to promote economical, social and environmental development worldwide through sustainable scenarios related to energy production [1]. However, saccharification and bioproduct manufacturing from lignocellulose biomass are complex and lengthy processes. The current schemes for the biotechnological conversion of plant cell wall polysaccharides rely on first reducing biomass recalcitrance through a pretreatment step, and afterward, enzymatic cocktails are needed to breakdown biomass into more simple, fermentable saccharides, which could be fed into several bioprocesses, such as bioethanol production.

Despite the advantages of enzyme-catalyzed processes, *i.e.*, speed, specificity and mildness, the high cost of enzyme production and low catalytic efficiency are still major hindrances for cellulosic bioethanol. Thus, relevant biotechnological challenges in this field include the improvement of the catalytic efficiency of enzymes, the economic benefit and the synergy between the type of pretreatment and enzymatic load, and reduction of the cost of enzyme production by filamentous fungi [2], [3].

The enzymatic degradation of cellulosic materials by fungal enzyme systems, especially those produced by *Tricoderma reesei* and *Aspergillus* spp., has been extensively studied due to its effectiveness for the liberation of fermentable sugars for bioethanol production [4], [5]. Several efforts, such as optimization of fermentation processes [6, 7, and 8] and genetic modifications of the microorganisms [9, 10, 11, and 12], are being targeted for improvement of fungal enzymatic systems. More recently, *Penicillium echinulatum* has been the focus of attention due to its potential to produce large amounts of cellulases at low costs; it has also been considered a promising strain for the bioethanol industry [7, 8, 9, 10, 13 and 14].

Advances in proteomics analysis have pushed forward secretome studies on filamentous fungi [15, 16, 17 and 18], mainly because they can highlight pathways as well as target genes for deletions or co-expression, to improve strains for biotechnological purposes [19]. In addition, secretome studies are appealing for basic research regarding the role of filamentous fungi not only as ubiquitous saprophytes in nature but also as cell factories to secrete sizeable amounts of proteins [20]. In the case of cellulolytic fungi, secretome analyses focus on describing the glycoside hydrolases (GH) and accessory components involved in the degradation of plant cell wall polysaccharides, the expression of which depend on the genetic background and culture conditions [8, 17 and 21].

Lignocellulosic biomass as a resource for bioenergy may have significantly lower environmental impacts than coal or other fossil fuels [22], [23]. However, to gain competitiveness over thermochemical processes, technological improvements are still necessary to make the bioconversion route profitable. Comprehending the enzymatic apparatus of cellulolytic strains, with a focus on achieving better efficiency and cost of enzyme production, is a key biotechnological bottleneck. To that end, our study presents the first global characterization of the secretome of a *P. echinulatum* cellulase-hyper-producing strain, which was subjected to different cultivation regimes, including integral sugar cane bagasse and pretreated lignocellulosic biomasses. Our strategy consisted of shotgun proteomic analysis of the fungal secretome followed by comprehensive chemical characterization of the biomass used as the source of fungal nutrition and biochemical activity assays using a collection of natural polysaccharides. Taken together, these approaches allowed better comprehension of the degradative capability of an industrial cellulolytic strain.

## Methods

### Fungal Strain and Growth Conditions

*P. echinulatum* strain 9A02S1-DSM18942 [10] was grown in PDA (Potato Dextrose Agar) for up to 7 days at 29°C until conidia formed. Mycelia suspension cultures were then grown in liquid medium [24], supplemented with cellufloc and kept on a shaker for 24 hours at 200 rpm and 29°C to serve as inoculum. Then, five different fermentation conditions were performed. The fermentation media were inoculated with 10% (v/v) of the above-mentioned culture in Erlenmeyer flasks containing 200 mL of a media containing 1 g/L peptone, 10% saline solution (KH_2_PO_4,_ 20 g/L; (NH_4_)_2_SO_4,_ 14 g/L; urea, 3 g/L; MgSO_4_•7H_2_O, 3 g/L; CaCl_2_, 3 g/L; FeSO_4_•7H_2_O, 0.05 g/L; ZnSO_4_•7H_2_O, 0.014 g/L; MnSO_4_•H_2_O, 0.016 g/L; CoCl_2_, 0.02 g/L) and carbon source at 10 g/L. Five different types of biomass were used as carbon sources: microcrystalline cellulose powder (MCL) (Celuflok 200 - Celuflok, São Paulo, Brazil), sugar cane bagasse derived from sulfuric acid treatment (SAT), bagasse derived from steam-explosion treatment (SET), bagasse derived from hydrothermal treatment (HDT) and integral sugar cane bagasse (SCB). After 5 days of growth at 29°C and 200 rpm, the biomass/mycelia was harvested from the supernatant. The supernatants derived from these five different biological experimental conditions were used for secretome analysis and the resultant pellets of these experiments were used for biomass decay composition analysis.

Briefly, the pretreated bagasses were prepared as described below:

- SAT: Integral bagasse was treated in a 1% H_2_SO_4_ solution over a 20-min residence time at 120°C using a solid-liquid ratio of 1∶10 (g/mL) in a 350-L reactor of stainless steel 316 L.
- SET: Integral bagasse was subjected to steam-explosion treatment over a 7-min residence time at 200°C in a custom-made 5 m^3^ reactor of stainless steel 316 L.
- HDT: Integral bagasse was subjected to hydrothermal treatment over a 10-min reaction time at 190°C in a REGMED reactor (model AU/E-20).

### Biomass Composition

Cellulose, hemicellulose, lignin and phenolic compounds in the five substrates (SAT, SET, HDT, SCB and MCL) were determined according to Gouveia *et al.* [25]. Representative samples of 200 mg were hydrolyzed in two steps: 72% H_2_SO_4_ for 7 min at 45°C followed by dilution to 3% H_2_SO_4_ and an additional 30 minutes at 121°C. The samples were then quenched in ice and filtered. The cellulose and hemicellulose contents of the filtrates were determined by high-performance anion exchange-pulsed amperometric detection chromatography (HPAEC-PAD) with a Dionex ICS-3000 (Thermo Fisher Scientific, Sunnyvale, CA, USA) system using a CarboPac PA 10 column (4×250 mm Dionex, Sunnyvale, CA, USA). The monosaccharide contents found in the hydrolysates were converted to percentage of polysaccharides. Finally, the quantity of total phenolic residue was determined by summing the acid-soluble and acid-insoluble phenolic fractions measured spectrophotometrically and gravimetrically. All these analyses were performed in triplicate.

### Mass Spectrometry Analysis (LC-MS/MS)

Culture filtrate aliquots from each experimental condition (100 µg of protein, determined by the Bradford method) were denatured in 8 M urea (1∶1) and reduced in 5 mM dithiothreitol (DTT) for 30 min at 56°C and alkylated in 14 mM of iodoacetamide (IAA) for 30 min in darkness at room temperature. Another addition of 5 mM DTT for 15 min was performed to quench unreacted IAA. The urea was reduced to a final concentration of 1.6 M by adding water, and 1 mM CaCl_2_ was added to serve as a trypsin co-factor. The proteins were digested overnight using 50 µl of trypsin (20 µg/µl Porcine Prep Grade Sequence, Promega - Madison, WI, USA) or 60 µL of α-chymotrypsin (20 µg/µl) (Promega- Madison, WI, USA) at 37°C and 30°C, respectively. The reaction was stopped by adjusting to pH 2 with trifluoro acetic acid and then centrifuged for 10 min at 2500×*g*. The peptide mixtures were desalted on a C18 Sep-Pak cartridge (Waters Associates Inc., Milford, MA, USA) according to the manufacturer’s instructions and dried in a Speed-Vac (Eppendorf - Hauppauge, NY, USA).

Each sample was resuspended using 12 µl formic acid (0.1%), and an aliquot (4.5 µL) of the resulting peptide mixture was separated by C18 (100 µm×100 mm) RP-nano UPLC (nanoAcquity, Waters) coupled with a Q-Tof Ultima mass spectrometer (Waters Associates Inc., Milford, MA, USA) with nano-electrospray source at a flow rate of 0.6 L/min. Two different UPLC gradient conditions were performed, using from 2 to 90% acetonitrile in 0.1% formic acid over 60 min or 90 min. The instrument was operated in the ‘top three’ mode in which one MS spectrum is acquired followed by MS/MS of the top three most-intense peaks detected. The spectra were acquired using MassLynx v.4.1 software, and the raw data files were converted to a peak list format (mgf) without summing the scans by the Mascot Distiller v.2.3.2.0, 2009 (Matrix Science Ldt.) and searched against the NCBI Database (11346708 sequences; 3868708671 residues; Version 2011; Fungi Taxonomy:678749 sequences) using the MASCOT v.2.3.01 (Matrix Science Ltd.) search engine with carbamidomethylation as a fixed modification, oxidation of methionine as a variable modification, one trypsin missed cleavage and a tolerance of 0.1 Da for both precursors and fragment ions. Only peptides with a minimum of six amino acid residues that showed significant threshold (p<0.05) in Mascot-based score were considered in the results [26]. The false positive identification was minimized using a cutoff corresponding to a protein confidence level of 95% (FDR of <1.0%).

We performed a label-free quantitation approach and the determination of the relative abundance of peptide ions was based on the number of unique peptides. According to Moi *et al.* (2012), there is a positive linear correlation between the emPAI and the number of unique peptides (r  = 0.741, p<0.0001) for analysis of protein composition among different biological treatments [27].

### Enzyme Activity Assay of Culture Supernatants

The standard enzymatic assay for biomass hydrolysis evaluation was performed in triplicate following Cota et al. [28], where 50 µl of substrate solution (0.5% w/w polysaccharide in water) was added to 40 µl of sodium acetate buffer (pH 5.0) and 10 µl of crude enzymatic extract (supernatant from fungal culture). The mixture was incubated at 50°C for different periods (min) depending on the polysaccharide nature. A set of 11 complex polysaccharides and 2 ρ-nitrophenol substrates (ρNP) were used (purchased from Megazyme - Wicklow, Ireland and Sigma–Aldrich - St, Louis, MO, USA). The reaction incubation times were 10 min for potato starch, β-glucan, laminarin, pectin, debranched arabinan and xyloglucan; 20 min for beechwood and birchwood xylans, lichenan, larch arabinogalactan, sugar beet, galactomannan and mannan; and 60 min for CMC, ρNP -cellobiose, and ρNP-glucose. It was utilized polysaccharides in both the linear and branched forms, such as potato starch (α-1-4∶1-6 glucose linked), laminarin from *Laminaria digitata* (β-1-3∶1-6 glucose linked), lichenan from *Iceland moss* (β-1-3∶1-4 glucose linked), carboximethylcellulose (β-1-4 glucose linked), debranched arabinan (α-1-5 arabinose linked), sugar beet (α-1-5∶1-3∶1-2 arabinose linked), pectin from citrus peel (α-1-4-D-galacturonic acid linked), galactomannan and mannan (mainly β-1-4 mannose linked), xylan from beechwood and birchwood (β-1-4 xylose linked). The enzymatc activity and substrate specificity were determined from the amount of reducing sugar liberated from different polysaccharide substrates by the dinitrosalicylic method [29] or ρNP liberated from *ρ*-nitrophenol substrates. One unit of enzyme activity was defined as the quantity of enzyme that released reducing sugars or ρNP at rate of 1 µmol/min/mg of protein. All these analyses were performed in triplicate.

## Results

### Chemical Analysis of Biomass Decay after Fungal Growth

**Figure 1:**
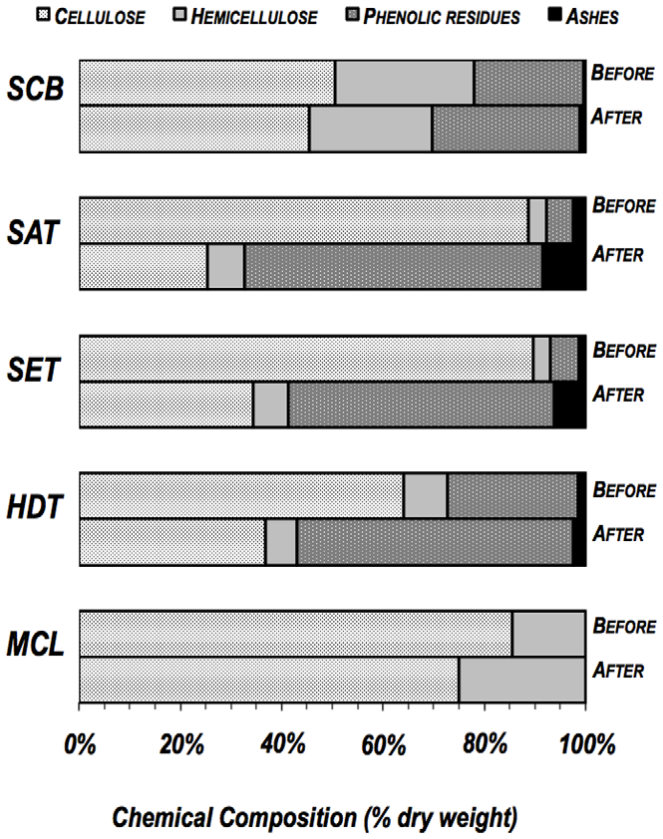
Chemical composition of the substrates derived from sugar cane bagasse (SCB, SAT, SET and HDT) and cellulose (MCL) before and after *P. echinulatum* growth.

**Figure 2:**
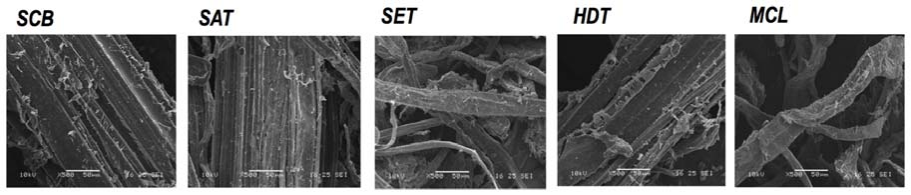
Scanning electron microscopy of substrates used for fungal growth. Samples of substrates derived from sugar cane bagasse (SCB, SAT, SET and HDT) and cellulose (MCL) were lyophilized (LabconcoFreezone 6 Benchtop) and stored at −80°C. The lyophilized samples were coated with a thin layer of gold in a sputter coater and examined in a JSM-5900 LV scanning electron microscope at acceleration voltages of 10 kV.

This study explored the inherent capacity of some filamentous fungal strains to produce biomass-conversion enzymes upon growth on lignocellulosic substrates. Four lignocellulosic substrates, four derived from sugar cane bagasse, including integral SCB, three different pretreated sugar cane bagasses (SET, SAT and HDT), and commercial cellulose (MCL) were used for fungal growth. Despite the significant differences in chemical composition observed in all substrates used in this study (Figure 1), all of the substrates presented cellulose, followed by hemicellulose, as a major component. Lignin was an abundant component only in HDT (26%) and in CBG (22%). Despite the similarity in between SET and SAT regarding plant structural polysaccharide content, scanning electron microscopy showed that these two biomasses differ significantly from each other physically (Figure 2).

As observed in Figure 1, the percentage of macromolecules (cellulose, hemicelluloses and lignin) in the pretreated sugar cane bagasse (SAT, SET and HDT) drastically changed after fungal growth. The relative content of cellulose of these substrates decreased by 71% for SAT, 62% for SET, 43% for HDT, 12% for MCL and 10% for SCB. The relative amount of xylan also changed for all substrates after *P. echinulatum* growth (Figure 1). The hemicellulose content of SCB and HDT decreased by 17% and 29%, respectively. In contrast, for SET, SAT and MCL, the relative xylan content did not decrease after five days of growth. Under these conditions, the xylan content augmentation was possibly the outcome of superior cellulose intake by the fungus and a lower initial content of hemicellulose in these substrates. Nevertheless, the findings collectively suggest that *P. echinulatum* consumes both cellulose and hemicelluloses but prefers cellulose for its development and growth. Accordingly, the relative amount of phenolic residues, along with ash content, increased under all conditions tested.

### Profiling of the P. echinulatum Secretome for Lignocellulose Degradation

**Figure 3:**
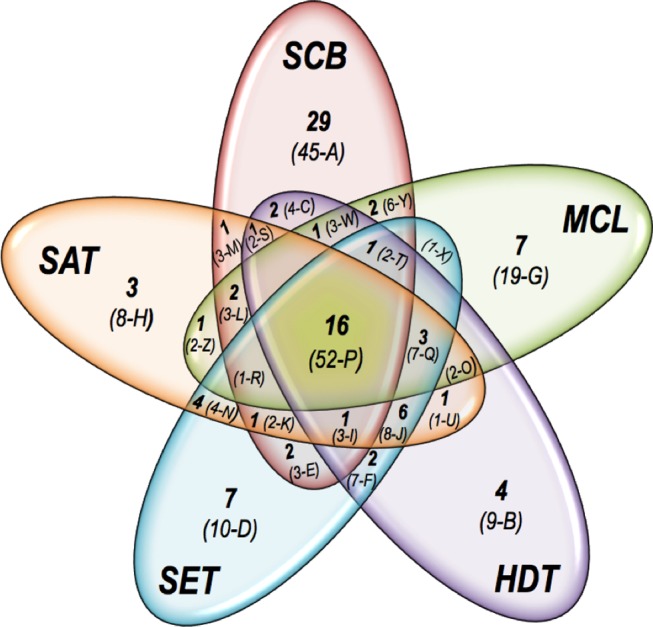
A Venn diagram describing the number of protein matches and peptides shared by the different secretomes analyzed. The number of protein matches identified in each condition is shown in bold. In parenthesis, the number of unique peptides matched in each condition and the peptide reference in Tables S1, S2 and S3 are indicated.

The main focus of this study was to reveal the largest possible number of secreted proteins involved in lignocellulose degradation by *P. echinulatum*. We combined several approaches aiming at to achieve better results of protein discovery. Five different types of biomass were used as carbon sources for fungal nutrition. The supernatants derived from the five biological experimental conditions were used for secretome analysis. A secretome sample from each biological condition was digested with trypsin or chymotrypsin, and two different LC-MS/MS operating running conditions were performed. Accordingly, each biological sample was evaluated four times by LC MS/MS. Collectively, 20 secretome analyses were performed. For each secretome sample analyzed, not all the same proteins were identified in the four LC MS/MS runs due mostly to different protocol preparations, but it was evident that the identification of proteins were improved in terms of both number of different proteins and number of peptides in each experimental design (data not shown). This strategy allowed the identification of 99 fungal proteins with a total 206 unique peptides (including the same sequence with different charge states) (Figure 3). In total, 16 proteins (identified from 52 unique peptides) were identified under all growth conditions, 29 proteins (45 unique peptides) were identified only in the secretome derived SCB, 7 proteins (11 unique peptides) from growth on SET, 3 proteins (8 unique peptides) from growth on SAT, 4 proteins (9 unique peptides) from growth on HDT and 7 proteins (19 unique peptides) from growth on MCL (Figure 3). The complete list of protein matches, peptide sequences with their respective *m/z* values are presented in Tables S1, S2, S3 and S4.

**Figure 4:**
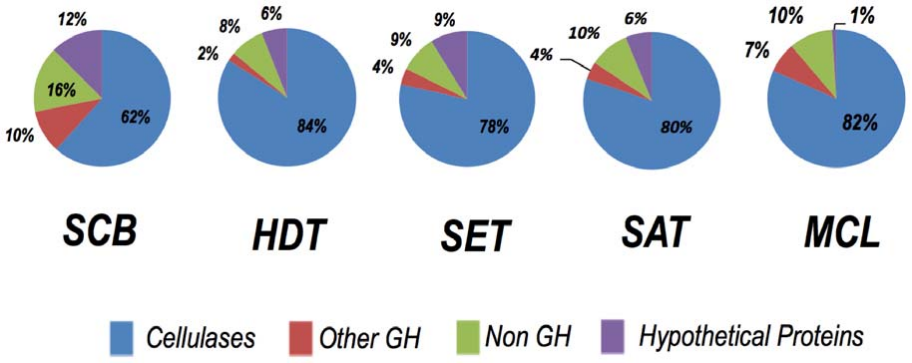
The distribution of protein matches identified in each growth condition with the respect to their classification in CAZy families of cellulases (Blue), hemicellulases and other GH family members (Red), proteins of predicted function (Green) and hypothetical proteins (Purple).

Most of the matches (∼90%) were to proteins with predicted functions (derived from 182 unique peptides including the same sequence with different charge states) (Tables S1 and S2). Of the proteins identifications with putative function, the majority (74%) belonged to families in the CAZy database (Table S1), which is a specialized database for structurally related enzymes that degrade, modify or create glycosidic bonds [30]. Of these protein matches, β-glucosidases (GH3), endoglucosidases (GH5, GH12, GH17 and GH61) and exoglucanases (GH6 and GH7) collectively represented more than 80% of the protein matches in all secretomes, except in the SCB secretome where matches to these GH families totaled 62% (Figure 4). Thus, the enzymatic repertoire of *P. echinulatum* is geared toward cellulose-degrading enzymes (Figure 4).

**Figure 5:**
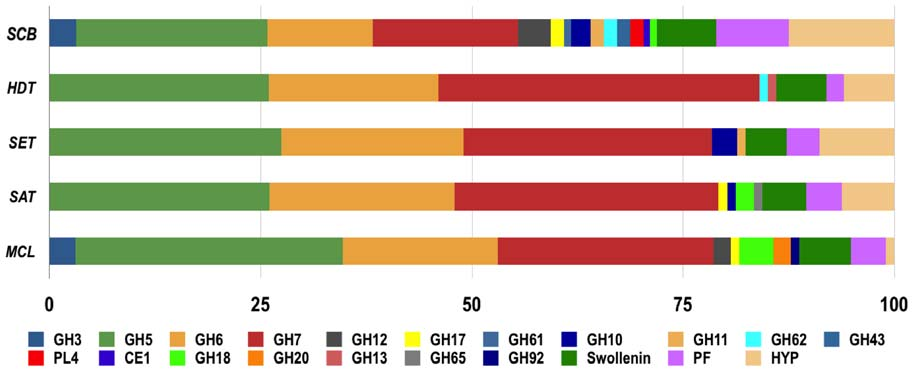
Distribution of peptide ions with similarity to CAZy enzymes, proteins with predicted function (PF) and proteins with hypothetical function (HYP). The tandem mass spectrometry analyses of the *P. echinulatum* secretome allowed the unambiguous assignment of 206 unique peptides, which were distributed across 18 CAZy families. The bar size indicates the percentage of total peptide matches in each condition.

While matches to GH3 (β-glucosidases) and GH12 (endoglucanases) were found only in the secretomes derived from fungi cultivated on SCB and MCL, matches to GH5 (endoglucosidases), GH6 and GH7 (exoglucanases) were found in all growth conditions (Figure 5). A match to a GH61 family member, a copper metalloenzyme that enhances cellulase activity [31], was identified only in the SCB-grown secretome (Figure 5). Two unique peptides with matches to GH17 family members, which includes endo-1,3-β glucosidades (EC 3.2.1.58) and licheninase (EC 3.2.1.73) activities, were identified in the secretome derived from fungi grown on SCB, MCL and SAT (Figure 5).

Matches to other CAZy family members were also observed in our study (Figure 5). Protein matches for xylanases, including GH10 and GH11, were mainly identified in the secretome derived from fungi grown on SCB. Peptides with matches to xylanases were identified in the secretome derived from fungi grown on SCB (6 unique peptides), SET (4 unique peptides) and SAT (1 unique peptides) (Figure 5; Table S1). Four unique peptides were accounted for β-galactosidases and arabinanases, which are included in the GH43 and GH62 families (Figure 5). Two unique peptides from rhamnogalacturonase (GH28), which is a pectinolytic component, as well as one peptide match for acetylxilan esterase (CE2), were identified only in the secretome derived from fungi grown on SCB (Table S1). Peptide identifications to chitinases (GH18) were identified in the secretomes derived from fungi grown on SCB, SAT and MCL (Figure 5). Amylase (GH13), *N*-acetylglucosidase (GH20) and trehalase (GH65) were identified in the secretomes from fungi grown on HDT, SAT and MCL, respectively (Figure 5). One peptide match to α-mannosidase (GH92), which is protein class potentially involved in protein deglycosylation, was identified in the secretome derived from fungi grown on MCL (Table S1).

Most of the matches to non-CAZy members (proteins of putative function or hypothetical proteins) were single peptide hits (Tables S2 and S3); however, some protein identifications are worth further mention. Matches to swolenins were identified under all growth conditions (Figure 5) and constituted 5% of the total number of unique peptides (including the same sequence with different charge states) observed in this study (Table S2). In addition, protein identification to a short-chain dehydrogenases/reductases family member from *A. oryzae* (gi|169774933) was identified with the confidence criterion of two unique peptides from different peptide sequences in the secretome derived from *P. echinulatum* grown on MCL; this protein contains a peptide signal for secretion (Table S2), according to SignalP (http://www.cbs.dtu.dk/services/). The hypothetical protein from *Trichophyton verrucosum* (gi|291189059), which is predicted to enter the non-classical secretory pathway according to Secretome P (http://www.cbs.dtu.dk/services/), was consistently found in all analyzed secretomes (Table S3).

### Functional Analysis of the P. echinulatum Secretome

**Figure 6:**
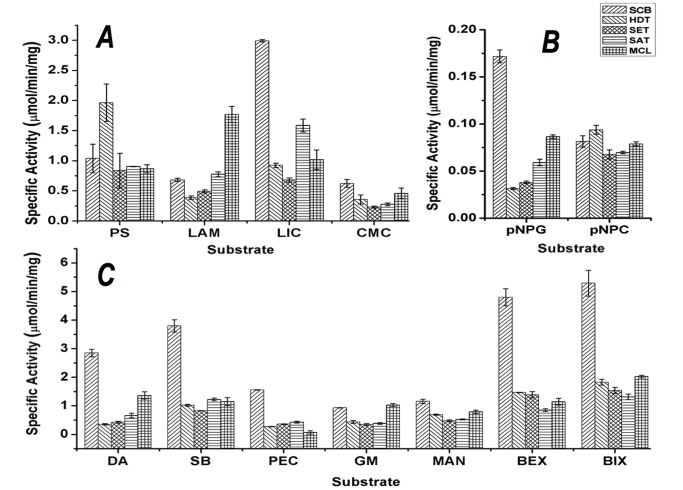
Specific enzymatic activities (µmol/min/mg) of *P. echinulatum* secretomes derived from five growth conditions (SCB, HDT, SET, SAT and MCL) against several polysaccharides: potato starch (PS), laminarin (LAM), lichenan (LIC), carboxymethylcellulose (CMC), p-nitrophenyl-D-gluco-pyranoside (pNPG), p-nitrophenyl-D-cellobioside (pNPC), debranchedarabinan (DA), arabinanform sugar beet (SB), pectin (PEC), galactomannan (GM), mannan (Man), beechwoodxylan (BEX), birchwoodxylan (BIX).

To further validate and describe the enzymatic repertoire available for plant polysaccharide degradation in *P. echinulatum*, hydrolytic performance was biochemically assessed using a set of 11 natural polysaccharides and two ρNP substrates. Supernatants derived from cultures grown under all five conditions were able to hydrolyze all glucose-containing polysaccharides tested, including glucose β-1,4 linked polysaccharide (CMC), β-1,4 and β-1,3 linked glucan (lichenan), β-1,3 and β-1,6 linked glucan (laminarin) and glucose linked with α-1,4 bonds (Starch) (Figure 6-A). The *P. echinulatum* secretome hydrolyzed ρNP-cellobiose and ρNP-glucose, highlighting the cellobiohydrolase and β-glucosidase activities of this strain (Figure 6-B) [8]. The fungus secretome also efficiently hydrolyzed hemicellulose and pectin, including xylan from birch and beech wood, mannan, galactomannan, arabinan (either linear or branched types) and citrus pectin (Figure 6-C).

**Figure 7:**
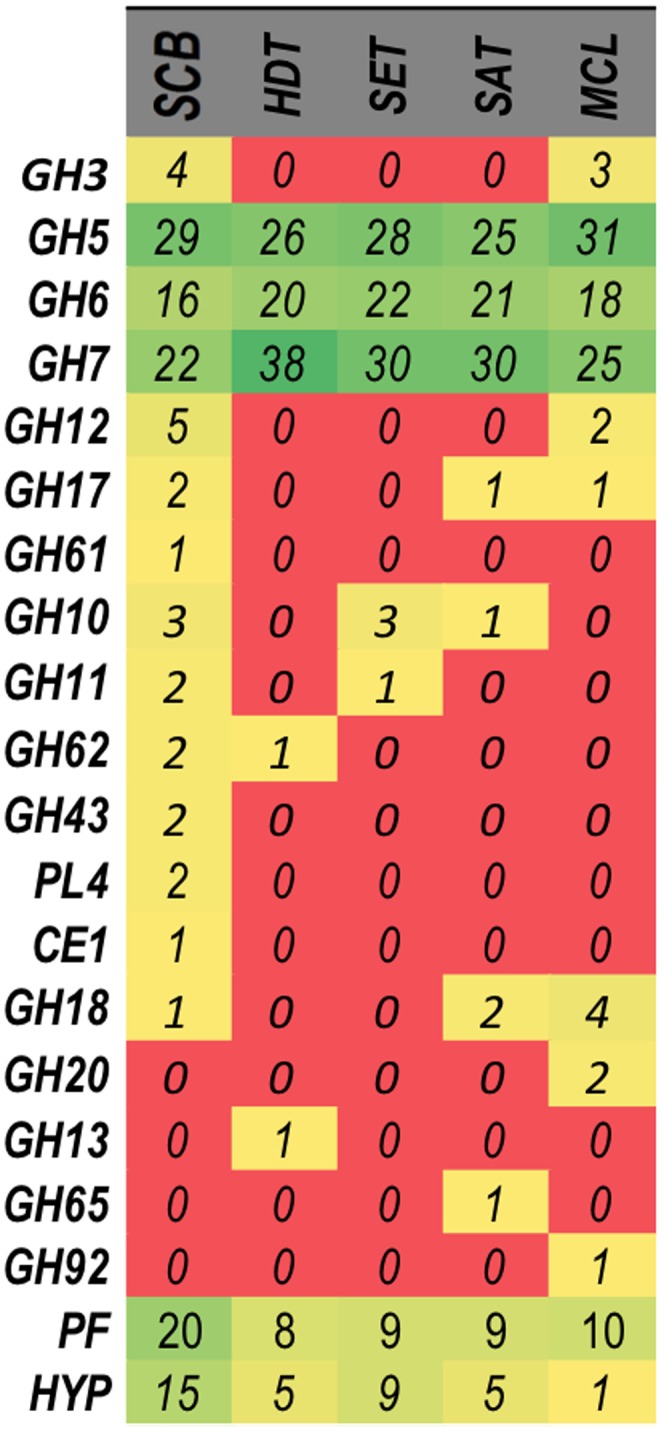
Heat map showing the relative abundance of the unique peptide ions in the different secretomes analyzed. Rows of the heat map correspond to the protein families, and columns correspond to the carbon sources used for fungal growth: SCB, HDT, SET, SAT and MCL. The peptide matches were categorized based on their similarity to CAZy families, proteins with predicted function (PF) and hypothetical proteins (HYP). The color of each protein families related to unique peptides matches abundance in the different growth conditions, varying from a minimum (red) to maximum (green) abundance.

The proteins assigned for each condition to specific CAZy families was in agreement with the biochemical analysis (Figure 6 and Figure 7), thereby validating the proteomic results obtained in this study. For example, GH3 (β-glucosidases) matches were assigned only in secretomes produced on SCB and MCL (4 and 3 unique peptides, respectively; Figure 6), an observation that correlates with the greatest specific activity on ρNP-glucose (a specific substrate for β-glucosidases) evidenced by these two secretomes (Figure 6-B). The higher number of peptide matches to GH5 and GH12 (endoglucosidases) in SCB- and MCL-grown secretomes was also in agreement with biochemical results for CMC (the chosen substrate for endoglucanase activity evaluation). The SCB-grown secretome exhibited the greatest hydrolytic activity against xylan (both from birch and beech wood), citrus pectin and arabinan (both linear and branched types) (Figure 6-C), which correlates with the greater number of matches to GH10, GH11, GH28, GH43, GH62 and CE1 (Figure7). The highest enzyme activity on potato starch was from the HDT-grown secretome, where a GH13 match was identified (Figure 6-A and 7). Similarly, protein matches for GH17 were found only in SCB- and MCL-grown secretomes (Figure 7), the enzyme sources with greatest lichenase activity (Figure 5-A).

*P. echinulatum* supernatants also showed some enzymatic activity against other polysaccharides, such as mannan and galactomannan, which suggest the presence of β-mannosidases (Figure 6-C). However, β-mannosidases were not detected in our proteomic analysis. Furthermore, proteomic data did not explain the significantly higher laminarin degradation activity observed in the MCL-grown secretome (Figure 6-A). A greater diversity of GH family members and a superior number of non-CAZy proteins (Figure 6) were identified in the secretome produced by fungi grown on SCB, which was the most complex and recalcitrant substrate used for fungal growth (Figure 1). However, 26% of the matches found in SCB-grown secretome had no peptide secretion signal, suggesting that some proteins were released after cell lysis or by an alternative secretion pathway (data not shown).

## Discussion

Microbial cellulase and hemicellulase production is dependent on the carbon source [32]. The substrates used in this study ranged from well defined, such as crystalline cellulose, to heterogeneous and complex, such as SCB, in composition and structure. Comprehensive chemical analysis of the lignocellulosic feedstock and the decayed biomass after fungal growth were performed. This analysis showed that the biomass composition drastically changed after fungal growth and suggested that *P. echinulatum* efficiently consumed both cellulose and hemicellulose.

We tested four types of sugar cane bagasses, “in natura” bagasse and steam exploded, hydrothermally and acid-treated. We hypothesized that by using a diverse set of substrates for fungal cultivation, it would disclose a broader view of the lignocellulose-degrading capacity, thus we expected to reveal the largest possible number of secreted proteins of *P. echinulatum.* Two main reasons have driven us to select those substrates. The first reason was to challenge *P. echinulatum* to different substrate composition and/or nutritional availability of carbon source. The second reason for selecting the five different types of biomass was to evaluate the *P. echinulatum* competence on producing glycoside hydrolases after its cultivation on cheap substrates, such as Cellufloc and sugar cane bagasse. Cellufloc is a consistent cellulose carbon source used for *P. echinulatum* enzyme production and it is well established for its cultivation [10]. The sugar cane bagasse is a readily available feedstock in the sugar and bioethanol industry, therefore a potential carbon source for industrial cellulases production.

Filamentous fungi have myriad industrial applications and for this reason they have been the focus of many biotechnological researches. This is especially true for *Penicillium* species that had a historical importance since the discovery of penicillin. An efficient way to evaluate the biotechnological potential of a fungus is to analyze its secretome. *P. chrysogenum* had its secretome recently described [33] and it was shown that, besides the presence of relevant enzymes for food industry, such as sulfydryl oxidase, dihydroxy-acid dehydratase, or glucoamylase, 12% of its secretome is devoted to plant cell wall degradation. Several previous secretome studies of *Ascomycetes,* such as *Aspergillus* [20] and *Trichoderma* [34], and basiomycetes, such as *Phanerochaete chrysosporium* [35] and *P. carnosa* [15], based on two-dimensional gel electrophoresis (2-DE) are available. The secretome of *P. funiculosum* was analyzed by both 2-DE gels and shotgun proteomics, and according to the authors, the shotgun methodology has improved the number of protein identifications [36]. Recently, a similar shotgun strategy described the enzymatic repertoire involved in plant polysaccharide degradation by the lower termite *Coptotermes gestroi* digestome [37].

In this study, nearly one hundred proteins were identified, the majority of which could be directly correlated with the degradation of lignocellulosic biomass. The identified GHs with potential relevance to cellulose digestion included the following families: 3, 5, 6, 7, 12, 17 and 61. In the *T. ressei* secretome, cellobiohydrolases have been reported as the most abundant secreted enzymes [38], [39], [40]. Our findings suggest that this class of enzyme is also highly secreted by *P. echinulatum*. In contrast, simply one peptide match to a GH61 endoglucanase (Cel61A) was identified in the secretome derived from fungi grown on SCB. GH61 enzymes have a positive influence on the activity of other cellulases [41] by introducing chain breaks and oxidized chain ends into cellulose polymers [42]. Our proteomic approach identified peptide matches to swollenin in all five experimental growth conditions. Rubin et al. [43] also previously identified swollenin cDNA from random sequencing of a *P. echinulatum* cDNA library. Swollenin are expansin-like proteins that have been proposed to disrupt hydrogen bonds between cellulose microfibrils or between cellulose and other cell wall polysaccharides [44].

We identified hemicellulolytic and pectinolytic components of the *P. echinulatum* secretome, such as GH10 and GH11 xylanases (identified from fungi grown on SCB, SET and SAT), GH43 and GH62 α-arabinofuranosidases (identified only from fungi grown on SCB), GH28 rhamnogalactoronases (identified only from fungi grown on SCB) and CE1 acetylxylan esterases (detected only from fungi grown on SCB). These results evidenced that the SCB promoted the secretion of a broader repertoire of biomass conversion enzymes. Regarding the composition, SCB was the most heterogenic and complex substrate tested in our study, and supposedly the most recalcitrant [45] compared to the other pretreated sugarcane bagasses and MCL.

The secretome analysis presented herein for *P. echinulatum* are in agreement to previous studies of other *Penicillium* species [8], [36], [46], which reinforces the capacity of this group of filamentous fungi to efficiently produce cellulases. According to Krogh et al. [46], the FPA activity was higher for *P. brasilianum* IBT 20888 than *T. reesei* Rut C30, which is a good diagnostic for cellulolytic capacity. Guais et al. [36] described a enzymatic cocktail termed “Rovabio™ Excel derived from *P. funiculosum,* as well as, evidenced that the endo-glucanases, cellobiohydrolases and xylanases were the most abundant proteins. Moreover, our results indicated that *P. echinulatum* is a cellulolytic fungus, likewise *T. reesei*. Adav et al. [34] evidenced the hypercellulolytic nature of *T. reesei* by identifing 65 cellulolytic enzymes, 37 hemicellulolytic and 34 lignin-degrading proteins. Other fungal species, such as *A. nidulans* [47] and *Fusarium verticillioides* [48], have their secretome more specialized to degrade hemicellulose rather than cellulose. The secretome analysis of *P. echinulatum* did not reveal components of the ligninolytic system such as peroxidases, which suggests that its secretome differentiate from the one from *Phanerochaete chrysosporium*, which is well known by its lignin and cellulose degrading capacity [49].

The biochemical secretome profiling of *P. echinulatum* confirmed that the enzymatic repertoire of this fungus is devoted to cellulose degrading enzymes. In addition, the approach conducted in this study allowed the validation of the protein identification results with both high- and low-confidence criteria. The sum of unique peptide sequences assigned to specific CAZy families for the different growth conditions was in agreement with the biochemical analysis. Both proteomic and biochemical analyses underlined the broader diversity of enzymatic activities when SBG was used as substrate for fungal growth. However, the present secretome analysis failed to identify extracellular oxydases (laccase, manganese peroxidase) or proteases. This failure may be due to the low concentration of these proteins and the incomplete *P. echinulatum* genome sequence. The use of more sensitive LC-MS/MS equipment with higher accuracy, such as Orbitrap mass analyzers, would generate larger data sets, and further studies may be required to reveal additional components, such as lignin-degrading enzymes and oxidases.

Lignocellulosic biomass pretreatment results on chemical composition and physical structure changes of the substrate, including redistribution of the hemicelluloses and lignin fractions, increasing surface area and formation of channels and pores [50]. Our results suggested that the enzyme production by *P. echinulatum* varied as a response to different substrate composition and/or nutritional availability of carbon source. For instance, the use of pretreated sugarcane bagasse as a source for *P. echinulatum* nutrition improved the production of cellulose degrading enzymes, such as GH5, GH6 and GH7 (endo and exo-glucanases). Whereas, the use of CBG as substrate, a broad enzymatic repertoire was disclosed, which included hemicellulose and pectin degrading enzymes.

The outcomes described in this study should contribute to the designing of optimized enzyme mixture for efficient conversion of sugarcane bagasse into fermentable sugars. Our findings pointed at the possibility of modulating fungal enzyme production and the development of tailor made enzymatic cocktails for different conversion pathways or biotechnological applications. Finally, the analysis presented herein is the first to show a global picture of the enzymatic repertoire for plant polysaccharide-degrading enzymes produced by *P. echinulatum.* This fungus has several biotechnological applications, including biomass to bioethanol. These findings will provide the biochemical and molecular basis for further studies on *P. echinulatum*, from which a number of applied technologies for biofuels production can be devoted.

## Supporting Information

Table S1 **CAZy enzymes identified by LC/MS-MS from supernatants of** ***P. echinulatum*** **grown on five different substrates (SCB, HDT, SET, SAT and MCL).** The table includes the information of the protein match and the respective accession number, peptide sequence, mass-to-charge ratio (m/z) and the presence/absence of signal peptide, along with the specific condition that the peptide ion was identified. The signal peptide prediction was analyzed using the signal peptide prediction program SignalP version 3.0 (http://www.cbs.dtu.dk/services/SignalP), for classical secretion signal motifs) and SecretomeP, for nonclassical signal motifs (25, 26). It is indicated (E) whether the peptide was derived from digestion with trypsin (T) or chymotrypsin (C). The reference to Figure 2 for each peptide match is also included.
(XLSX)

Table S2 **Proteins with predicted function identified by LC/MS-MS from supernatants of** ***P. echinulatum*** **grown on five different substrates (SCB, HDT, SET, SAT and MCL).** The table includes the information of the protein match and the respective accession number, peptide sequence mass-to-charge ratio (m/z) and the presence/absence of signal peptide, along with the specific condition that the peptide ion was identified. The signal peptide prediction was analyzed using the signal peptide prediction program SignalP version 3.0 (http://www.cbs.dtu.dk/services/SignalP), for classical secretion signal motifs) and SecretomeP, for nonclassical signal motifs [51], [52] (25, 26). It is indicated (E) whether the peptide was derived from digestion with trypsin (T) or chymotrypsin (C). The reference to Figure 2 for each peptide match is also included.
(XLSX)

Table S3 **Hypothetical proteins identified by LC/MS-MS from supernatants of** ***P. echinulatum*** **grown on five different substrates (SCB, HDT, SET, SAT and MCL).** The table includes the information of the protein match and the respective accession number, peptide sequence mass-to-charge ratio (m/z) and the presence/absence of signal peptide, along with the specific condition that the peptide ion was identified. The signal peptide prediction was analyzed using the signal peptide prediction program SignalP version 3.0 (http://www.cbs.dtu.dk/services/SignalP), for classical secretion signal motifs) and SecretomeP, for nonclassical signal motifs [51], [52] (25, 26). It is indicated (E) whether the peptide was derived from digestion with trypsin (T) or chymotrypsin (C). The reference to Figure 2 for each peptide match is also included.
(XLSX)

Table S4 **The unique proteins identified at each specific growth condition.** The protein classification was based on the similarity to CAZy enzymes (GH families), proteins with predicted function (PF) and proteins with hypothetical function (HYP).
(XLSX)
